# Fungal 7-*epi*-10-deacetyltaxol produced by an endophytic *Pestalotiopsis microspora* induces apoptosis in human hepatocellular carcinoma cell line (HepG2)

**DOI:** 10.1186/s12906-017-1993-8

**Published:** 2017-11-28

**Authors:** Kamalraj Subban, Satpal Singh, Ramesh Subramani, Muthumary Johnpaul, Jayabaskaran Chelliah

**Affiliations:** 1 Department of Biochemistry, Indian Institute of Science, Bangalore, Karnataka 560012 India; 2 Centre for Advanced Studies in Botany, University of Madras, Guindy Campus, Chennai, Tamil Nadu India; 3 Department of Biology, College of Engineering, Science & Technology, Fiji National University, Natabua Campus, Lautoka, Fiji Islands

**Keywords:** Endophyte, *Pestalotiopsis*, 7-*epi*-10-deacetyltaxol (EDT), Apoptosis, G2/M arrest

## Abstract

**Background:**

Paclitaxel (taxol) is a potent anticancer drug that is used in the treatment of a wide variety of cancerous. In the present study, we identified a taxol derivative named 7-*epi*-10-deacetyltaxol (EDT) from the culture of an endophytic fungus *Pestalotiopsis microspora* isolated from the bark of *Taxodium mucronatum*. This study was carried out to investigate the effects of fungal EDT on cell proliferation, the induction of apoptosis and the molecular mechanisms of apoptosis in human hepatoma HepG2 cells in vitro.

**Methods:**

The endophytic fungus was identified by traditional and molecular taxonomical characterization and the fungal EDT was purified using column chromatography and confirmed by various spectroscopic and chromatographic comparisons with authentic paclitaxel. We studied the in vitro effects of EDT on HepG2 cells for parameters such as cell cycle distribution, DNA fragmentation, reactive oxygen species (ROS) generation and nuclear morphology. Further, western blot analysis was used to evaluate Bcl-2-associated X protein (Bax), B-cell lymphoma 2 (Bcl-2), p38-mitogen activated protein kinase (MAPK) and poly [ADP-ribose] polymerase (PARP) expression.

**Results:**

We demonstrate that the fungal EDT exhibited significant in vitro cytotoxicity in HepG2 cells. We investigated cytotoxicity mechanism of EDT in HepG2 cells. The results showed nuclear condensation and DNA fragmentation were observed in cells treated with fungal EDT. Besides, the fungal EDT arrested HepG2 cells at G2/M phase of cell cycle. Furthermore, fungal EDT induced apoptosis in HepG2 cells in a dose-dependent manner associated with ROS generation and increased Bax/Bcl-2 ratio, p38 MAPKs and PARP cleavage.

**Conclusions:**

Our data show that EDT induced apoptotic cell death in HepG2 cells occurs through intrinsic pathway by generation of ROS mediated and activation of MAPK pathway. This is the first report for 7-*epi*-10-deacetyltaxol (EDT) isolated from a microbial source.

## Background

Taxol, also known as paclitaxel, is a complex diterpenoid compound originally reported from bark of the Pacific yew tree, *Taxus brevifolia* [1] and later reported in other yew species [2]. Taxol is widely used against breast, ovarian and lung cancers [3, 4]. Acute supply crisis prevails for paclitaxel as chemotherapeutic drugs since its concentration in yew bark is exceedingly low and extraction process is complicated and expensive [5]. Besides, overexploitation of yew bark has led to serious diminishing of *Taxus* forests [6, 7]. Therefore, several approaches have been utilized for increasing taxol accessibility and finding alternative sources through chemical synthesis, tissue and cell cultures of the *Taxus* spp. [8–12]. However, the efforts failed to increase the yield of taxol, improve the complicated process and decrease the cost [8, 11, 13]. This finally compelled the researchers to explore the microbial world. Microbial fermentation with the benefits of optimization of fermentation conditions and co-cultivation offers suitable inexpensive method of choice to increase yield of taxol production. In the microorganisms, taxol was first reported from an endophytic fungus *Taxomyces andreanae* isolated from the inner bark of *Taxus brevifolia* [14]. A large number of taxol-producing endophytic fungi such as *Pestalotiopsis microspora*, *Taxomyces andreanae*, *Fusarium* spp., *Alternaria* sp. and *Tubercularia* sp. have been reported from *Taxus* plants since then [15–20]. Additionally, several reports have shown that non-*Taxus* plants also harbour taxol-producing endophytic fungi such as *Periconia* sp., *Bartalinia robilldoides* and *Pestalotiopsis guepinii* [21–23]. A total of 100 reports of endophytic fungi belonging to 72 fungal species from 32 different host plants have been reported so far for taxol production [24].

Cancer is one of the leading causes of death in the world [25] and hepatocellular carcinoma (HCC) is the fifth most common cancers worldwide and the third most common reason for cancer-related mortality [26]. Surgical resection and liver transplantation are inefficient for advanced HCC [27, 28]. Hence, it is imperative to develop new therapeutic drugs with high efficacy and low toxicity for HCC. Apoptosis, a programmed cell suicide, is usually a physiological event that does not induce inflammation [29]. Therefore, apoptosis induction is considered a desired therapeutic goal in cancer treatment to reduce possible adverse side effects [30]. Many studies have demonstrated apoptosis by taxol treatment in diverse cancer cells including breast cancer, glioblastoma, hepatoma and ovarian cancer. Taxol triggers apoptosis by diverse pro-apoptosis stimuli converging on mitochondria, causing mitochondrial depolarization and caspase enzymes activation eventually leading to apoptotic cell death [31–38]. In the course of continuous research on plant-fungus associations and in search of novel bioactive secondary metabolites from endophytic cultures, a taxol derivative, EDT obtained from an endophytic fungus *P. microspora* associated with *T. mucronatum* is being reported herewith. It is the first studies to report EDT from a microbial source. We also report characterization and comparison of anti-proliferative and apoptosis inducing activity of EDT in hepatocellular carcinoma cells (HepG2), as well as investigate the molecular mechanisms triggering apoptosis.

## Methods

### Isolation and identification of endophytic fungi from *T. mucronatum*

The fungus used in this study was one of 27 endophytic fungi isolated from the inner bark of *Taxodium mucronatum* obtained in Ootacamund, South East India. The voucher specimen was deposited at Madras University Herbaria and Culture Collection in Centre for Advanced Studies in Botany, Chennai with accession number MUBL1013. The *T. mucronatum* bark was cut into pieces (~0.5 × 0.5 × 0.5 cm) and treated with 70% (*v*/v) ethanol, washed with sterilized water and the outer bark removed with a sterilized sharp blade. Small pieces of inner bark were placed on the surface of PDA medium supplemented with 150 mg L^−1^ chloramphenicol in Petri plates and incubated at 26 ± 1 °C in 12 h light/dark chamber. After several days, fungi were observed growing from the inner bark fragments in the plates. Individual hyphal tips of the various fungi were removed from the agar plates, placed on new PDA medium and incubated at 26 ± 1 °C for at least 2 weeks. Fungus culture was checked for purity and transferred to fresh agar plate by the hyphal tip method [15]. Fungus was identified based on the morphology of the fungal culture, the mechanism of spore production and the characteristics of the spores [39]. For molecular identification, DNA extraction and ITS PCR was followed as described earlier [40]. The universal primers ITS1 and ITS4 were used for amplification. The PCR product was sequenced in an AVI377 automated DNA sequencer. The ten most similar sequences in Genbank were found for sequence by means of BLAST search. Sequences were aligned by using CLUSTAL multiple sequence alignment and gaps were excluded. The most informative sequences were used to construct phylogenetic tree using maximum parsimony by MEGA 4 [41] and the *Amanita muscaria* used for as an out group of organism. The fungal spores and mycelia were preserved in 15% (*v*/v) glycerol at −70 °C.

### Fermentation, extraction and fungal EDT isolation

The *Pestalotiopsis microspora* used in this study was grown in 4 l Erlenmeyer flasks containing 1 l modified M1D medium [42]. Twelve mycelial agar plugs of 0.5 × 0.5 cm, were used as inoculum. The fungus was grown at 26 ± 1 °C in 12 h light/dark chamber. After 18 days of incubation, the entire culture (1 l) was passed through four layers of cheesecloth. The culture fluid was extracted with two equal volumes of dichloromethane and the organic phase was taken to evaporation under reduced pressure at 40 °C. The residue was dissolved in 1 ml methanol, and subject to TLC on a 0.25 mm (10 × 20 cm) silica gel plate developed in solvent system of chloroform/methanol (7:1, *v*/v) with authentic paclitaxel (Sigma, Cat. No. T-7402). After chromatography, the silica gel plate was sprayed with 1% vanillin-sulphuric acid (*w*/*v*) and visualized under UV fluorescence at 254 and 365 nm for confirmation for taxol/taxanes with appropriate relative front (R_*f*_) by comparing with the reference paclitaxel.

A total of 14.7 g residue was dissolved in 5 ml methanol suspended with ~ 25 g of silica gel for preparation of slurry (silica gel + sample). Then the dry slurry placed on a 2 × 45 cm column of silica gel (60–120 mesh) equilibrated with chloroform. Elution of the column was performed in a step-wise manner starting with 70 ml 100% chloroform followed by mixtures of chloroform/acetone at 95:5, 90:10, 85:15, 80:20 till 0:100 (*v*/v). A fraction having the corresponding chromatographic mobility as authentic paclitaxel was found from the 75:25 to 70:30 fractions. These fractions were combined and evaporated to dryness yielding 1.5 g of residues, which was further subjected to a second 1.5 × 30 cm column of silica gel (200–300) and eluted with dichloromethane/acetone (25:75, v/v). This eluted fraction exhibited on R_*f*_ which was identical to reference paclitaxel. Then, the fraction subjected for *in vacco* at 40 °C yielded yellow powder (11.79 mg).

### Spectroscopic analyses for identification of fungal EDT

Nuclear magnetic resonance spectroscopy (NMR) was done on fungal EDT preparation in a JEOL JNM-ECP 600 MHz instrument with the sample dissolved in 100% deuterated methanol. X-ray powder diffraction (XRD) was studied for EDT by coating on the XRD grid and the spectra were recorded by using Philips PW1830 X-ray generator operated at voltage of 40 kV and a current of 30 mA using Cu Kσ^−1^ radiation. Liquid chromatography-Electrospray ionization-tandem mass spectrometry (LC-ESI-MS) was performed on Thermo Finnigan Survey or HPLC with dual wavelength (UV) detector connected to Thermo LCQ Deca XPMAX-MS platform and analysed by Xcalibur software. The EDT was dissolved in methanol and was injected with a spray flow of 2 μl min^−1^ and a spray voltage of 2.2 kV. Fourier transform infrared spectroscopy (FTIR) was recorded using Perkin Elmer Spectrum one FTIR over the region 4000-400 cm^−1^.

### Cell lines and culture conditions

HepG2 cells (human liver carcinoma cell line) used for the experiments was obtained from National Centre for Cell Sciences (NCCS), Pune, India. The cells were grown as monolayers in Dulbecco’s Modified Eagle Medium (DMEM) supplemented with 10% FCS, 1 mM sodium pyruvate, 10 mM HEPES, 1.5 g ml^−1^ sodium bicarbonate, 2 mM 1^−1^ glutamine and antibiotics (10,000 U ml^−1^ pencillin and 10 mg ml^−1^ streptomycin). Cell stocks were maintained in 75 cm^2^ tissue culture flasks in a humidified atmosphere of 5% CO_2_ and 95% air at 37 °C. Cultures were maintained in the medium until the confluent growth was attained. A cell density of at 1 × 10^6^ cells was maintained at the time of treatment. For all in vitro assays, fungal EDT was dissolved in dimethyl sulfoxide (DMSO) to make a stock solution at 1.284 mM concentration, sterilized using a sterile 0.22 μm membrane filter and stored at −20 °C. From the stock, 256.8 μM/ml was taken and diluted with cell culture medium in 2-fold serial dilutions (128.4, 64.2, 32.1, 16.05, 8.02, 4.02, 2.0, 1.0 μM/ml) and 0.5% DMSO maintained as a control.

### Cell survival assay

The cytotoxicity of purified fungal EDT was measured using 3-(4,5-dimethylthiazol-2-yl)-2,5-diphenyltetrazolium bromide (MTT, Sigma-Aldrich) colorimetric assay. Briefly, cells (1 × 10^4^ cells/well) were seeded in 96-well plates. After complete adhesion, different concentration of fungal EDT (0–128.4 μM) were added and incubated further for 24 h at 37 °C. The treated cells were then incubated in fresh DMEM medium containing MTT (5 mg mL^−1^) at 37 °C. After 4 h, the supernatants were discarded carefully and DMSO was added to dissolve the formazan crystals. The absorbance at 570 nm was measured with a microplate reader (Bio-Rad, USA). The cell survival was determined by the MTT test as the percentage of the ratio of the absorbencies of treated and untreated (control cells).

### Determination of apoptosis

Apoptosis was determined using acridine orange-ethidium bromide (AO-EBr) dual staining method by microscopically visualizing condensed apoptotic nuclei from normal ones [43]. HepG2 cells (3 × 10^4^) were treated with different concentrations of fungal EDT seeded in 6-well plates and incubated for 24 h. Cells were then stained with 1:1 ratio of AO and EBr. After staining, the cells were immediately visualized using fluorescence microscope (Olympus, CKX41, Japan) at a magnification 40× using 450–490 nm filter. The number of cells showing features of apoptosis was counted as a fraction of the total number of cells present in a field.

### Determination of nuclear morphology

The nuclear condensation was determined by 4′,6-diamidino-2-phenylindole (DAPI) staining. HepG2 cells (5 × 10^4^ cells/well) cultured in 12-well plates were incubated with and without fungal EDT for 24 h. The cells were then fixed with 3.7% (*v*/v) paraformaldehyde, permeabilized with 0.1% Triton X-100 and stained with DAPI (1 mg ml^−1^ in PBS) [44]. After washing twice with PBS, cells were observed under fluorescence microscope (Olympus, BX51, Japan) at 10× magnification using 485 nm excitation and 535 nm emission filter sets. The apoptotic cells were identified by the presence of highly condensed chromatin or fragmented nuclei.

### DNA fragmentation analysis

The DNA fragmentation was studied as described earlier [44]. HepG2 cells were cultured in 60 mm dishes to 70% confluence prior to drug treatment. The cells (5 × 10^6^) were treated with different concentrations of fungal EDT for 24 h. After treatment, the cells were harvested by centrifugation at 1000×*g* for 5 min and washed with ice cold PBS. The genomic DNA was extracted from the HepG2 cells using QIAamp DNA Mini Kit (Qiagen, USA), analysed using 0.9% (*w*/*v*) agarose gel and electrophoresed at 2 V/cm for 16 h. The DNA present in the gels was visualized under UV light after staining with ethidium bromide (1 μg ml^−1^) and photographed using gel documentation system (Gene Flash, Syngene, Bioimaging, Kubota 2420, Tokyo).

### Apoptosis assay by Propidium iodide (PI) staining

HepG2 cells (3.5 × 10^6^) were seeded in 6-well plates and treated with different concentrations of fungal EDT at 37 °C for 24 h in CO_2_ incubator. Cells (2 × 10^6^) were fixed in 90% ethanol in PBS at 4 °C for analyzing DNA. After 12 h, the cells were centrifuged at 2000 rpm for 5 mins and the cell pellet was suspended in ice cold PBS. The cell suspension was then treated with propidium iodide along with RNAase (50 μg ml^−1^) for 30 min at 37 °C in CO_2_ incubator then stored in the dark at 4 °C. The red fluorescence of the individual cells was measured at an excitation wavelength of 540 nm and an emission wavelength at 610 nm in a FACSCalibur flow cytometer (BD Biosciences, CA). A minimum of 10,000 events were analyzed per sample using CellQuest software.

### Measurement of intracellular reactive oxygen species (ROS)

The intracellular ROS generation was detected using an oxidant sensitive non-fluorescent probe DCFH-DA that gets oxidized by intracellular ROS to its fluorescent derivative, dichlorofluorescein (DCF) [45]. HepG2 cells (8 × 10^6^ cells/ml) seeded in 96-well plates were treated with different concentrations of fungal EDT for 24 h, followed by 10 μM DCFH-DA addition and further incubated for 30 min. The cells were washed with PBS to remove the excess dye and measurements were done using spectro-fluorophotometer (Shimadzu, RF-5301PC, USA) with excitation and emission filters set at 485 ± 10 and 530 ± 12.5 nm, respectively. Fluorescent microscopic images were taken using blue filter (450–490 nm) (Olympus, CKX41, Japan).

### Western blot analysis

Proteins were isolated from control and fungal EDT-treated cells as described previously [46]. Bax, Bcl-2, p38 MAPK, PARP and β-actin protein expression was investigated using Western blot analysis. Briefly, cells in 6 well plates were harvested and washed with PBS. Cells were lysed in 100 μl lysis buffer (20 mM Tris–Hcl, pH 7.4, 150 mM NaCl, 1 mM EDTA, 30 μg/ml aprotinin, and 1 mM phenylmethylsulfonyl fluoride) followed by centrifugation at 1000 g for 5 min at 4 °C. The supernatants (cytosolic fractions) were saved and the pellets solubilized in the same volume of mitochondrial lysis buffer (50 mM Tris pH 7.4, 150 mM NaCl, 2 mM EDTA, 2 mM EGTA, 0.2% Triton X-100, 0.3% NP-40, 100 μM PMSF, 10 μg/ml leupeptin, 2 μg/ml aprotinin), kept on ice and vortexed for 20 min followed by pelleting at 10,000 g for 10 min at 4 °C and subjected to 12.5% poly acrylamide gel electrophoresis lane. A total volume of 40 μg of protein was loaded per lane. The separated proteins were blotted onto a PVDF membrane by semi-dry transfer (Bio-Rad, USA). After blocking with 5% non-fat milk in TBS, the membranes were then incubated with various antibodies: anti-Bax, anti-Bcl-2, anti-p38 MAPK, anti-PARP and anti-β-actin. The dilutions used were p38 (1:1000), Bcl-2 (1:500), Bax (1:1000), PARP (1:500) and β-actin (1:2000). After primary antibody incubation, the membranes were incubated with secondary antibody at a concentration of 1:2000. Then the membranes were washed with Tris-buffered saline and 0.05% Tween-20 thrice for 10 min interval, after extensive washes in TBST, the bands was visualized by treating the membranes with 3, 30-diaminobenzidine tetrahydrochloride (Western blot detection reagent, Sigma, USA). Densitometry was done using ‘Image J’ analysis software.

### Statistical analysis

All data are presented as the means ± standard deviation (SD) for at least three independent experiments, and analyzed for statistical significance using one-way analysis of variance using software SPSS 11.5. A *p*-value <0.05 was statistically significant.

## Results

### Identity of the endophytic fungus

**Fig. 1:**
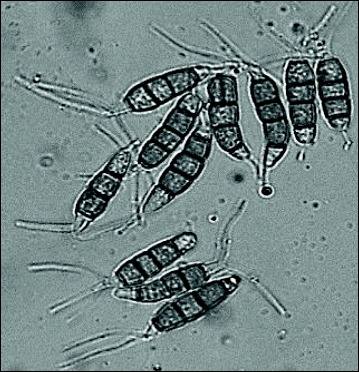
Spore morphology of *Pestalotiopsis microspora*

**Fig. 2:**
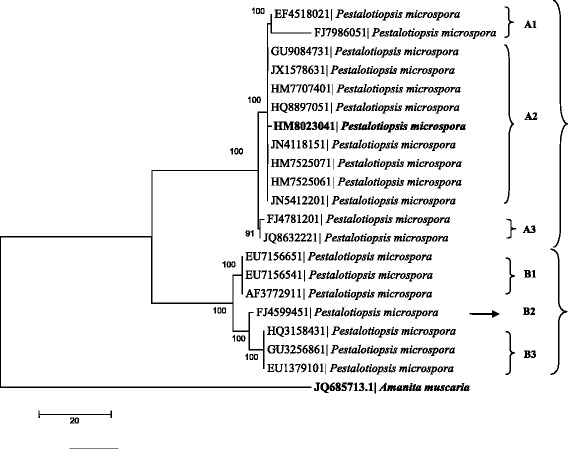
Maximum parsimony phylogram showing phylogenetic relationships among isolates of *Pestalotiopsis microspora* data were analysed with random addition sequence. Values in the branches are parsimony bootstrap equal or above 50%

The fungus used in this study was identified as *Pestalotiopsis microspora*. Conidia observed were clavate-fusoid, broad, tapering towards the base, 5-celled, straight, 15.69–29 × 6.73–9.5 μm; intermediate colored cells guttulate, amber or olivaceous, equally colored, lowest colored cell sometimes slightly paler, 15–20 μm long, slightly constricted at septa. Apical appendages 1–2; 5–6 μm, and basal appendage 1, 2.92–4.5 μm long (Fig. 1) as reported earlier [47]. It was further identified by molecular characterization using 18S rDNA sequence; which was deposited in Genbank database with an accession number of HM802304. Analysis of the ITS 28S rDNA sequence revealed 100% identity with *P. microspora*. The maximum parsimony phylogram showed phylogenetic relationships among isolates of *P. microspora*. Data were analysed with random addition sequence and their values in the branches were parsimony bootstrap (equal or above 50%) as shown in Fig. 2. The phylogeny of closely related species of *P. microspora* was investigated using individual and combined sequence analyses of nuclear ribosomal DNA (ITS rDNA). ITS ribosomal DNA based phylogenies indicate that all *P. microspora* strains including the holotype sequence (HM802304) constitute a strongly supported monophyletic clade (100%). Within this monophyletic clade, there are 2 subclades. In particular, it was noted that *P. microspora* are in different subclades sequences for which were collected from Genbank database except HM802304. They are from different places, conclusive relationships based on host associations of *P. microspora* is unwise as there are a number of isolates that have been isolated from different places. The host fungal relationships based on phylogeny have already been reported [48] and any association between hosts and species within *Pestalotiopsis* does not seem to be justified at present (Fig. 2).

### Fungal EDT from the *P. microspora*

**Fig. 3:**
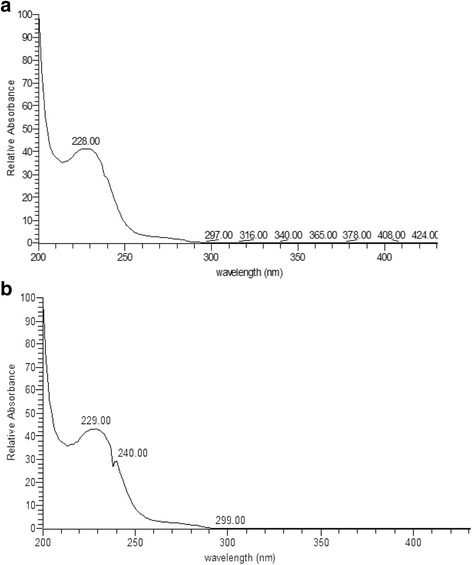
UV-visible spectroscopic analysis. **a** Fungal EDT (**b**) Paclitaxel

Fungal EDT having chromatographic properties comparable to paclitaxel in solvent system chloroform/methanol (7:1, *v*/v) which produced the same color reactions (bluish spot) with vanillin/sulfuric acid reagent and corresponding R_ƒ_ (0.80) with paclitaxel (0.78) under UV at 254 nm, was consistently isolated from *P. microspora*. Further, the fungal EDT isolated from *P. microspora* produced a maximum UV absorption spectrum at λ_max_ 228 nm distinguished from authentic paclitaxel, with a maximum at λ_max_ 229 nm (Fig. 3a, b).

**Fig. 4:**
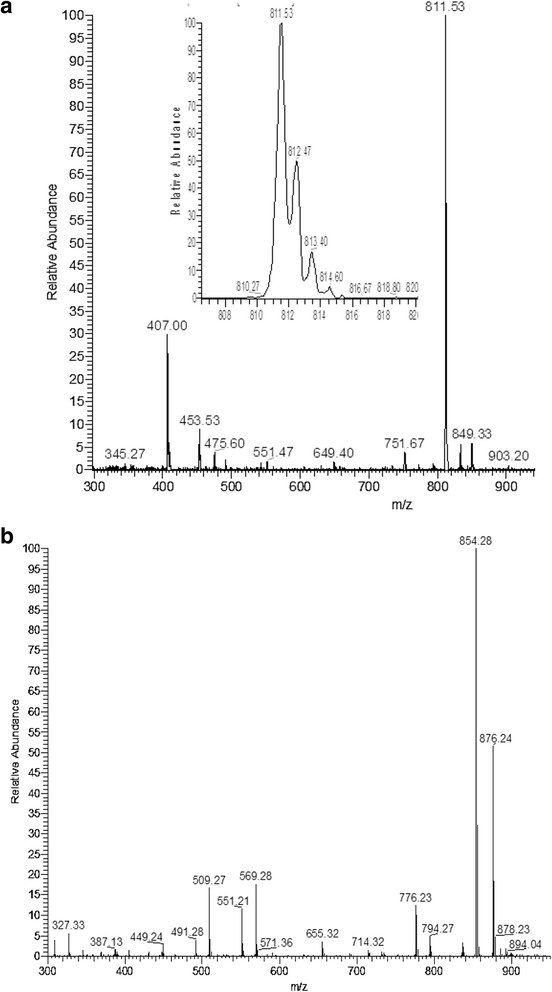
ESI-MS analysis of fungal EDT (**a**) MS- ESI spectrum of fungal EDT showing [M + H]^+^ *m/z* of 811.53, (**b**) ESI- MS spectrum of Paclitaxel showing [M + H]^+^ *m/z* of 854.28

**Fig. 5:**
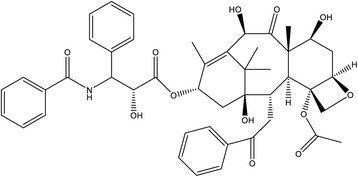
Structure of fungal 7-*epi*-10-de-acetyl taxol (EDT)

Using ESI-MS, ^1^H and ^13^C NMR and FTIR spectra, the structure of fungal EDT was deduced as 7-*epi*-10-deacetyltaxol, whose backbone protons have previously been assigned by [49–51] and their co-workers. As shown in Table 1, there is a clear match of our ^1^H NMR data with theirs. Additionally, we also assigned all carbon chemical shifts by ^13^C NMR and COSY (Table 2) along with data attributed by [49–51] and their co-workers. In fungal EDT, an acetyl group was absent however ^1^H–NMR spectrum displayed a marked peak at δ 4.77 ppm indicates the presence of hydroxyl group at C-10 (Table 1). Similar assignments were reported for plant-derived deacetyled taxol at C-10 position with a peak at δ 4.73 ppm in the ^1^H–NMR spectrum [51]. Further convincing spectroscopic evidence for the identity of fungal EDT was obtained by electrospray ionization-tandem mass spectroscopy. The fungal EDT yielded an (M + H)^+^ peak at 811.53 while authentic taxol yielded an (M + H)^+^ peak at 854.28 (Fig. 4a, b). The authentic paclitaxel differing by mass differences of 44 Da might indicate possible acetyl group with methine (C_2_H_4_O). Besides, the IR spectrum of the fungal EDT showed a broad absorption frequency in the range of 3451–3347 cm^−1^ attributed to the presence of hydroxyl (OH) and amide (NH) groups in addition to a broad peak between 1558 cm^−1^ and 1735 cm^−1^ for C = C stretching. Similar records have been reported for plant-derived 7-*epi*-10-deacetyltaxol [49]. Further, absorption frequency at 1005–1120 cm^−1^ indicated the presence of C-H bends. These results clearly showed that the fungal EDT is a taxol derivative of 7-*epi*-10-deacetyl taxol (Fig. 5). The fungal EDT structure was further confirmed by XRD analysis where five peaks at 2θ values 61.32, 38.0, 32.12, 29.9, and 21.0 deg. corresponding to (221), (101), (101), (011) and (001) planes of 7-*epi*-10-deacetyltaxol (EDT) were observed and compared with the Joint Committee on Powder Diffraction Standards (JCPDS), file No. No. 51–2319. The said 2θ values of five peaks are in accordance with the standard of JCPDS confirms/ indicates that the resultant compound is 7-*epi*-10-deacetyltaxol.

**Table 1:** Comparison of ^1^H NMR data of fungal EDT with previously reported plant-derived 7-*epi*-10-deacetyltaxol and 10-deacetyltaxol chemical shifts (δ in ppm) and coupling constants (*J*, Hz)

H	Fungal 7-*epi*-10-deacetyltaxol	7-*epi*-10-deacetyltaxol (Jun et al. 2001)		10-deacetyltaxol (Mclaughlin et al. 1981)		7-*epi*-10-deacetyltaxol (Zhang et al. 2010)	
	δ_H_	δ_H_	*J*, Hz	δ_H_	*J*, Hz	δ_H_	*J*, Hz
2	5.78 (d)	5.726(d)	*J* = 7.44 Hz				
5	4.88 (dd)	4.892(dd)	*J* = 4.09, 9.06 Hz				
6	2.38, 2.28	2.32, 2.287					
7	3.60 (s)	3.659 (s, broad)					
7-OH	4.77(d)					4.73 (d)	*J* = 12.2 Hz)
0	5.65 (s)	5.415 (s)					
10-OH	4.11					4.11(s, broad)	
13	6.23 (t)	6.228 (t)	*J* = 8.55 Hz				
14	2.29, 2.38	2.233, 2.36					
16	1.09 (s)	1.072 (s)					
17	1.18 (s)	1.183 (s)					
20	4.42	4.411, 4.382 (AB)	*J* = 8.55 Hz				
23	2.43 (s)	2.496 (s)					
C-2 OBz	7.59 (m), 8.18 (d)			7.53 (m), 8.18 (d)	*J* = 2,8 Hz		
OAc	2. 52 (s)			2.51 (s)			
2’	4.78 (s, broad)	4.78 (s)					
3’	5.78 (d)	5.796 (d)	*J* = 5.80, 9.00 Hz				
3′-Ph	7.46 (m)			7.40 (m)	*J* = 9 Hz		
3′-NBz	7.44 (m)			7.43 (m)	*J* = 2, 6 Hz		
*m/p*	7.34 (s)					7.37/7.36

**Table 2:** Comparison of ^13^C NMR data of fungal EDT with previously reported plant-derived 7-*epi*-10-deacetyltaxol, 10-deacetyltaxol and 7-*epi*-taxol chemical shifts (δ) and coupling constants (*J*, Hz)

Carbon No.	Fungal 7-*epi*-10- deacetyltaxol	7-*epi*-10-deacetyltaxol (Jun et al. 2001)	10-deacetyltaxol (Mclaughlin et al. 1981)	7-*epi*-taxol (Chmurny et al. 1992)
	δ_C_	δ_C_	δ_C_	δ_C_
1	78.683		78.90	
3	39.956	40.303		
5	68.097		68.04 (d)	
10	77.687	77.889		
12	132.346		132.06 (s)	
13	73.459	73.256		
15	44.713		43.54	
17	26.679	26.017		
18	14.098	14.419		
19	9.208		9.49 (q)	
20	77.434			77.6
21	167.779	167.108		
23	22.952	22.572		
2’	73.459	73.256		
3a	128.784	128.706		
4b	131.021	131.98		
q-Ph2	138.540			138.1
C33	168.890			167.2
C4-O-C OCH3	174.573			172.4

### Cytotoxic effect of fungal EDT on human hepatoma HepG2 cells

**Fig. 6:**
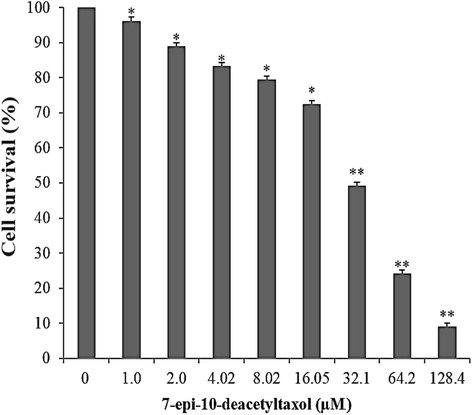
Cytotoxicity of fungal EDT against human hepatoma HepG2 cells. Each IC_50_ value represents means ± SD of 3 independent experiments

To examine the cytotoxicity, HepG2 cells was exposed to fungal EDT at different concentrations for 24 h before MTT assay. The results showed that fungal EDT significantly inhibited the growth (cell survival) of HepG2 cell lines, with 50% inhibitory concentration (IC_50_) values in the range from 0 to 128.4 μM. HepG2 showed the highest sensitivity to fungal EDT with an IC_50_ value of 32.1 μM (Fig. 6). Therefore, we further investigated the anticancer effects of fungal EDT on hepatoma HepG2 cells and the underlying mechanisms.

### Induction of apoptosis by fungal EDT in HepG2 cells in vitro

**Fig. 7:**
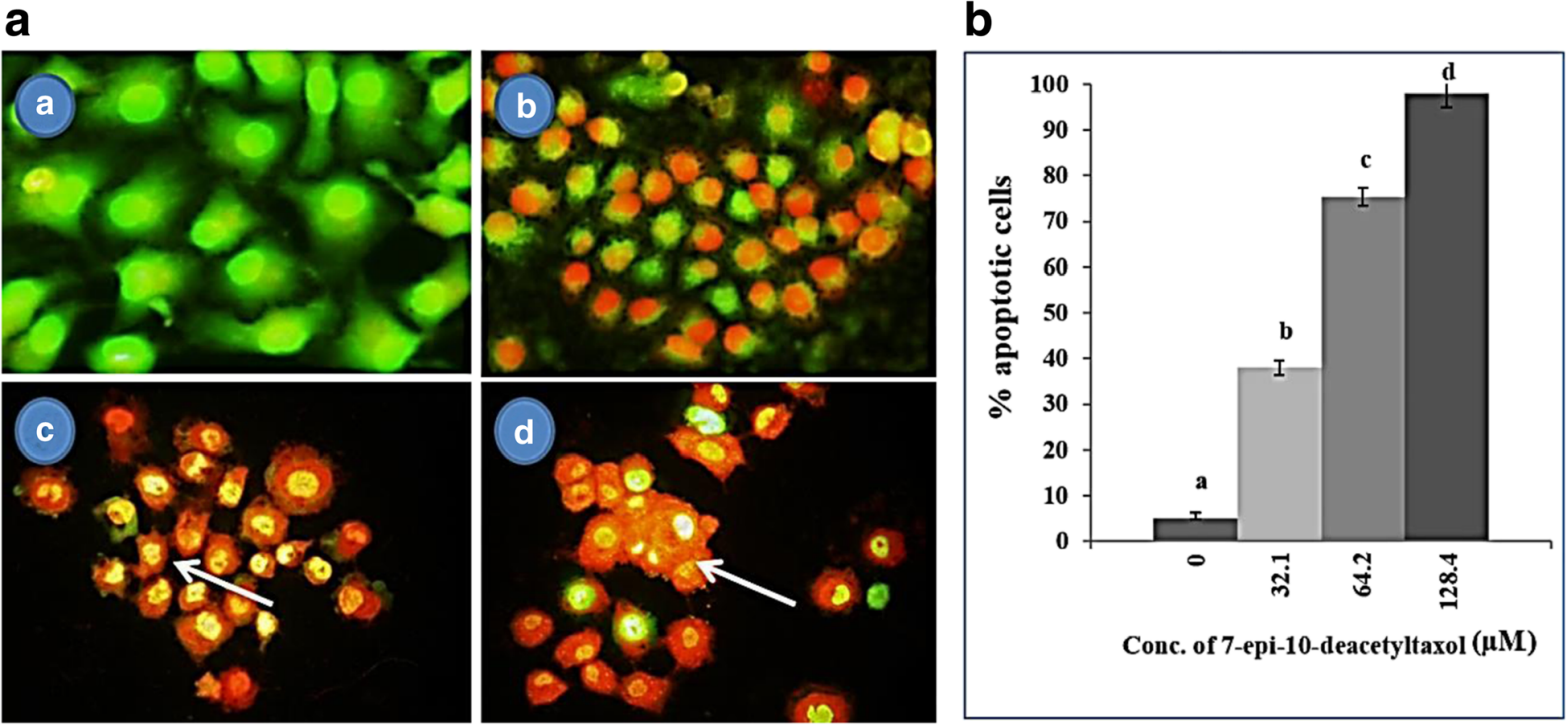
Fungal EDT induced apoptosis in HepG2 cells. **a** *A* Untreated control cells, (a *B-D*) HepG2 cells treated with 32.1 μM, 64.2 μM and 128.4 μM, respectively of fungal EDT. Fluorescence microphotographs of untreated and treated cells were obtained after AO/EB staining. Arrows pointing towards cells undergoing apoptosis, (**b**) The number of cells displaying apoptosis. Data are represented as means ± SD from 3 independent experiments

The effect of fungal EDT on cell apoptosis was evaluated by using AO-EBr dual staining method and was characterized by a reduction in green fluorescence in fungal EDT-treated cells in a dose-dependent manner. The control cells showed evenly distributed AO stain (green fluorescence) with no morphological changes whereas cells treated with fungal EDT displayed signs of apoptosis such as accumulation of red fluorescence and condensation nuclei (Fig. 7a). The percentage of apoptosis in HepG2 cells treated with 32.1, 62.4 and 128.4 μM of fungal EDT was 38.02, 75.36 and 98.03%, respectively (Fig. 7b).

### Fungal EDT induces changes in nuclear morphology

**Fig. 8:**
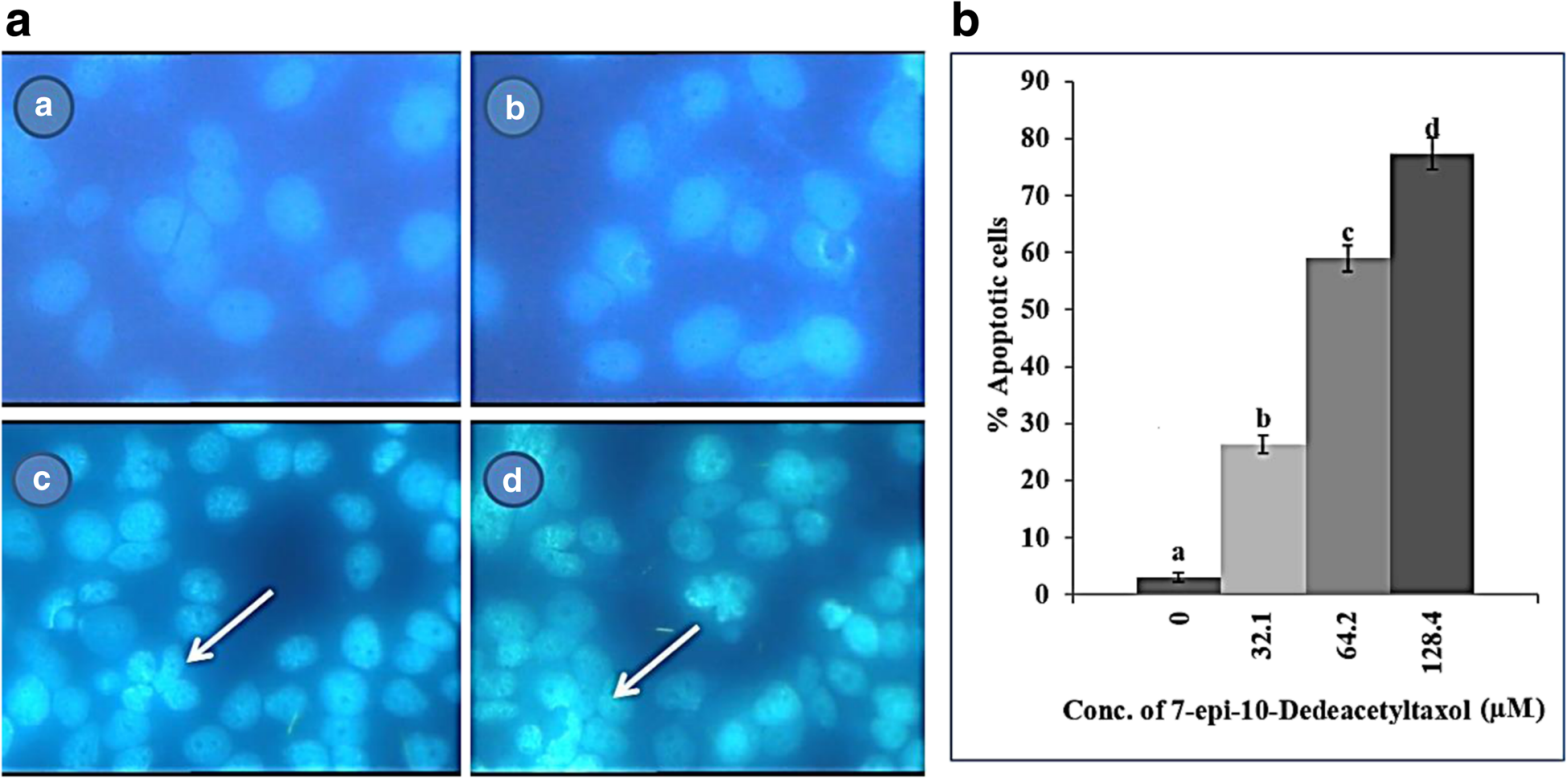
Effect of fungal EDT on nuclear condensation in HepG2 cells. Fluorescence microphotographs of untreated and treated cells were obtained after DAPI staining. **a** *A* Fluorescence microphotographs of untreated HepG2 cells, (a *B-D*) HepG2 cells treated with 32.1 μM, 64.2 μM and 128.4 μM, respectively of fungal EDT. Arrows indicating condensed nuclear foci. **b** The percentage of apoptosis in HepG2 cells treated with indicated concentrations of EDT

Changes in cell nuclear morphology, such as condensed and fragmented nuclei are considered late events of apoptosis. In order to identify the changes in cell nuclei in HepG2 cells upon treatment with various concentrations of fungal EDT, cells were stained with DAPI and visualized by fluorescence microscopy. The control cells displayed uniform fluorescence across the nuclei, while chromatin condensation in the form of punctuates foci nuclear pyknosis were observed in the cells treated with fungal EDT (Fig. 8a). The percentage of apoptosis in HepG2 cells treated with 32.1, 62.4 and 128.4 μM of fungal EDT was 26.32, 59.03 and 77.32%, respectively (Fig. 8b).

### Fungal EDT induce DNA fragmentation in HepG2 cells

**Fig. 9:**
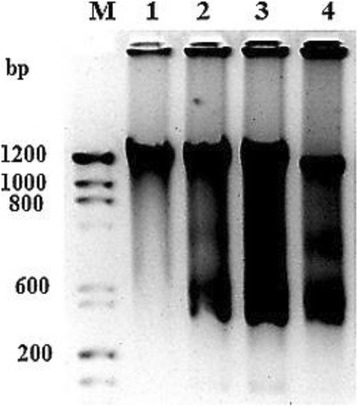
DNA fragmentation of HepG2 cells induced upon treatment with fungal EDT. M DNA molecular size marker, 1 DNA from untreated control cells, 2–4 show DNA from fungal EDT treated HepG2 cells with 32.1 μM, 64.2 μM and 128.4 μM, respectively

The fragmentation of nuclear DNA is one of the hallmarks of apoptosis. It is known that DNA fragmentation is carried out by the caspase activated DNase (CAD). Activation of CAD leads to cleavage of nuclear DNA into multiples of ~200 bp oligonucleosomal size fragments. To confirm the induction of apoptosis, HepG2 cells were treated with fungal EDT and nuclear DNA isolated from these cells was analysed in 0.9% agarose gel. DNA fragmentation was observed upon EDT treatment in HepG2 cells, while there is no DNA fragmentation seen in untreated cells (Fig. 9). This clearly confirmed that fungal EDT induced apoptosis in HepG2 cells.

### Determination of cell cycles by PI staining

**Fig. 10:**
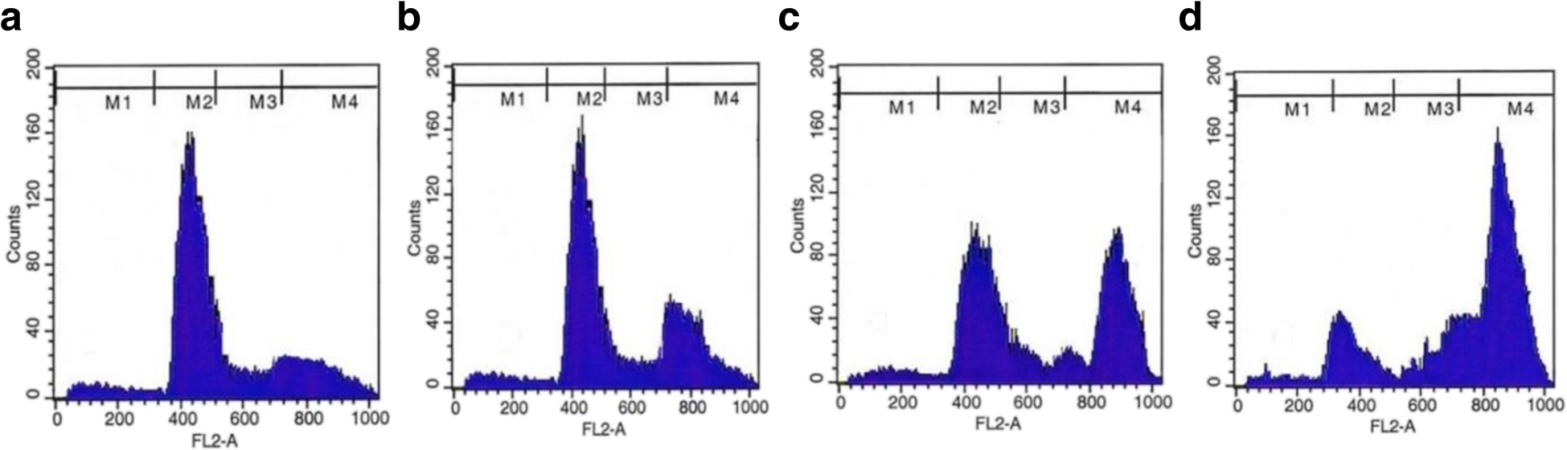
PI-FACS analysis of fungal EDT-treated HepG2 cells indicated that cells were arrested in the G2/M phases of cell cycle and subG1 phase with significant effect at 128.4 μM of EDT. **a** Untreated control cells, (**b-d**) HepG2 cells treated with 32.1 μM, 64.2 μM and 128.4 μM, respectively of fungal EDT

Flow cytometric analysis of apoptosis showed that fungal EDT induced apoptosis in HepG2 cells in a dose-dependent manner as shown in Fig. 10. At 32.1 μM fungal EDT a clear shift from G_1_ phase to G_2_/M was observed and the percentage of cells stalled at G_2_/M phase of cell cycle (Fig. 10) that confirmed by the PI staining.

### Effects of fungal EDT on intracellular ROS in HepG2 cells

**Fig. 11:**
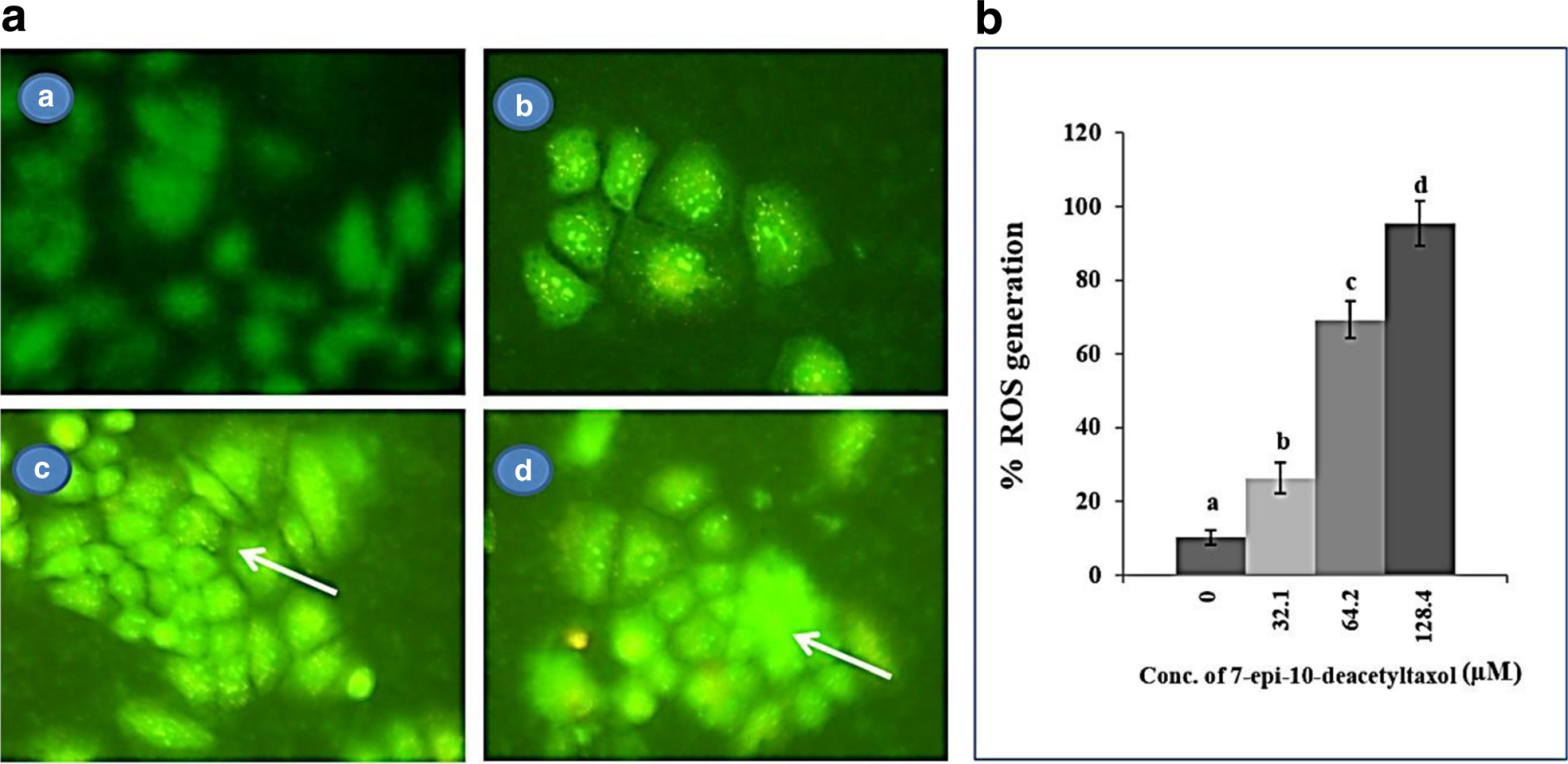
Fungal EDT induced intracellular reactive oxygen species (ROS) in HepG2 Cells. Photomicrographs of (**a** *A*) Untreated cells and (a *B-D*) HepG2 cells treated with 32.1 μM, 64.2 μM and 128.4 μM, respectively of fungal EDT. Arrows are pointing high level accumulation of intracellular ROS as DCF fluorescence, (**b**) Quantitative analysis of intracellular ROS by spectrofluorometery. Data are expressed as mean ± SD from three independent experiments

The fluorescence microscopy and spectrofluorimetry clearly established ROS induction in HepG2 cells by fungal EDT. DCFH-DA would be hydrolyzed to DCF in the cell and yields a highly green fluorescent signal in the presence of intracellular ROS (Fig. 11a). Quantitative analysis found that fungal EDT could rapidly increase the fluorescence intensity, indicating an increase in ROS level in HepG2 cells compared with the control cells about 10.07%. The mean intracellular DCF fluorescence intensity was increased by 25.92, 69.03 and 95.84% in HepG2 cells treated with 32.1 μM, 62.4 μM and 128.4 μM of fungal EDT, respectively (Fig. 11b). These results indicated that fungal EDT caused oxidative stress in HepG2 cells and ROS production plays an important role in fungal EDT-induced apoptosis.

### Effects of fungal EDT on the expression of apoptosis-related proteins in HepG2 cells

**Fig. 12:**
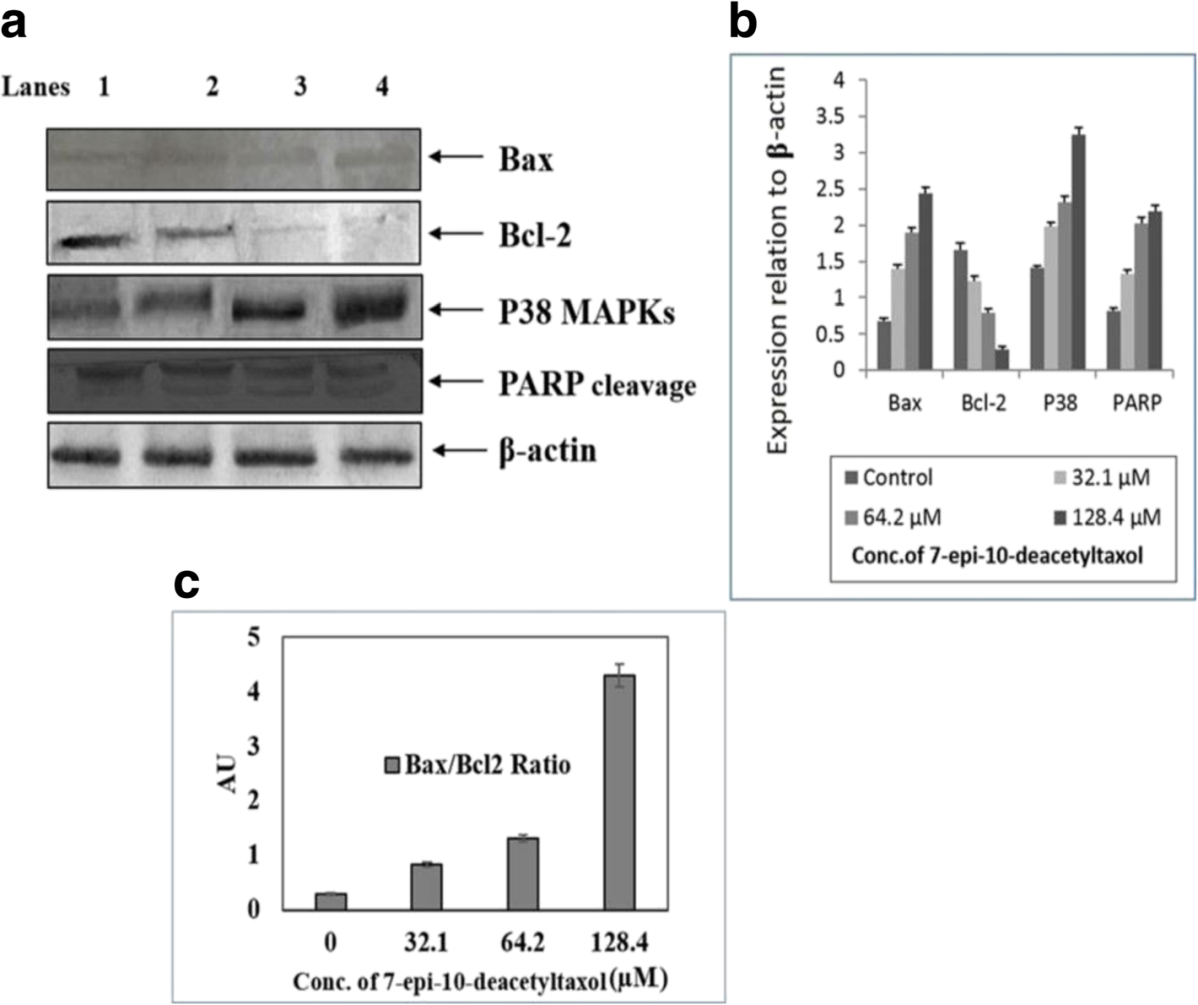
Effect of fungal EDT on the expression of apoptosis-related proteins in HepG2cells. **a** Qualitative expression profile of selected proteins by western analysis. Lane 1 Untreated control cells, Lane 2–4 HepG2 cells with 32.1, 64.2 and 128.4 μM, respectively of fungal EDT, (**b**) Quantitative analysis of expression of apoptosis-related proteins, (**c**) Histogram representing the ratio of pro-apoptotic Bax and anti-apoptotic Bcl-2 as a measurement of onset of apoptosis

In order to explore the mechanisms underlying fungal EDT induced apoptosis, the expression of apoptosis-related proteins in HepG2 cells was investigated by Western blotting (Fig. 12a–c). The characteristic apoptotic feature of PARP cleavage was observed in the treated cells starting from 32.1 μM (Fig. 12a). Meanwhile, the expression of pro-apoptotic protein Bax was increased while the anti-apoptotic protein Bcl-2 was suppressed by fungal EDT. The effects of fungal EDT on the expression of apoptosis-related proteins in HepG2 cells were in an obvious dose-dependent manner. As shown in Fig. 12b and c, the levels of p38, one of the major components of MAPK signalling pathways, was significantly increased in HepG2 cells treated with fungal EDT in a dose-dependent manner. The both quantitative and qualitative analysis of Bax/Bcl-2 ratio clearly indicates the apoptosis from the perspective of pro- and anti-apoptotic signal imbalance (Fig. 12a–c). These results further confirming the apoptotic inducing effect of fungal EDT in HepG2 cells.

## Discussion

The endophytic fungus, *Pestalotiopsis microspora* isolated from non-*Taxus* host *Taxodium distichum* was first reported in 1996 for taxol production [17]. At present, many endophytic fungi from *Taxus* /non-*Taxus* species including *Pestalotiopsis* spp. have been recognized for production of taxol and its derivatives [52]. In the present study, we report a taxol derivative, 7-*epi*-10-deacetyltaxol (EDT) isolated from the culture filtrate of *P. microspora* as confirmed by TLC and various spectroscopic/spectrometric analyses. To the best of our knowledge, this is the first report of isolation of EDT from only microbial source especially from *P. microspore. P. microspora* was identified based on morphological features and molecular data. The morphology of this strain is different from that of other taxol-producing endophytic *Pestalotiopsis* spp. [53–55]. The spores of *P. microspora* median cells have thicker walls and non-distoseptate [15–17, 47]. In ITS rDNA molecular characterization confirmed the strain as *P. microspora*.

The production of EDT by *P. microspora* to produce EDT was confirmed by isolation of a compound having chromatographic properties similar to authentic paclitaxel in solvent system chloroform/methanol (7:1, *v*/v) which showed a single dark bluish violet spot on TLC when sprayed with vanillin-sulfuric acid reagent and giving the corresponding R_ƒ_ (0.80) with paclitaxel (0.78) under UV fluorescence at 365 nm. The maximum UV absorption wavelength for fungal EDT was found (λ_max_ 228) identical to authentic taxol UV absorption at λ_max_ 229. Our result is coinciding with previously reported plant-derived 7-*epi*-10-deacetyltaxol [56, 57] in *Taxus* plants. In ESI-MS, molecular ions at m/z 811 attributing to the (M + H)^+^ and confirmed its molecular weight to be 810 for the fungal EDT [56, 57]. ^1^H and ^13^C NMR spectra were identical with the authentic taxol spectra and that of spectra previously reported for 7-*epi*-10-deacetyltaxol [49, 50, 58]. Further the functional groups in the fungal EDT analysed using FTIR showed peaks similar to those reported earlier for plant-derived 7-*epi*-10-deacetyltaxol [49]. In addition, to confirm the fungal EDT structure, XRD analysis was performed and the results showed closest match to 7-*epi*-10-deacetyltaxol verified by JCPDS computational database. Therefore, these results evidently display the fungal 7-*epi*-10-deacetyltaxol (EDT).

To study the anticancer activity of fungal EDT towards liver cancer cells, we used HepG2 cell line. Several studies have previously shown that taxol isolated from plants and endophytic fungi were effective in inhibiting cancer cell proliferation towards ovarian, breast and lung cancers [3, 4]. In the present study, fungal EDT significantly inhibited the growth of HepG2 cells, with an IC_50_ value as 32.1 μM for 24 h treatment, indicating that fungal EDT exhibits strong cytotoxicity against HepG2 cells. Induction of apoptosis is regarded as a novel therapeutic strategy for cancer treatment [29]. Many anticancer agents have been reported to induce death of tumor cells by triggering apoptosis [59]. In our results, fungal EDT strongly induced apoptotic cell death in HepG2 cells in a dose-dependent manner as evidenced by EtBr/AO staining, suggesting that the anticancer effect of fungal EDT on HepG2 cells was mediated through the induction of apoptosis. The nuclear shrinkage, chromatin condensation and fragmentation are the main hallmarks of apoptosis; these were observed upon treatment of the cell lines with fungal EDT at 62.4 and 128.4 μM. Inter-nucleosomal DNA fragmentation represents the terminal step in the events leading to apoptosis. Gel electrophoresis of the genomic DNA isolated from fungal EDT-treated HepG2 cells displayed the DNA fragmented. Similar DNA fragmentation was reported on paclitaxel exhibited cell viability on MCF-7 cells [20]. PI-FACS analysis of fungal EDT-treated HepG2 cells indicated that cell cycle arrested in the G2/M phase with significant effect at 64.2 μM and 128.4 μM arresting cells at 50.08 and 80.16%, respectively.

Several chemotherapeutic agents exert their anticancer effects through inducing the generation of ROS, and the intrinsic apoptotic pathway is especially susceptible to ROS [60]. In the present study, the production of intracellular ROS increased remarkably in HepG2 cells treated with fungal EDT, while inhibition of ROS production by the control cells significantly decreased the apoptosis, suggesting that fungal EDT-induced apoptosis in HepG2 cells was closely associated with the production of ROS, which may act as upstream signalling molecules to initiate mitochondria-mediated cell apoptosis. ROS is involved in the opening of the mitochondrial permeability transition pore, depolarization of the mitochondrial membrane, and then the release of mitochondrial pro-apoptotic factors in the process of mitochondria mediated apoptosis [61, 62]. Fungal taxol isolated from *F. solani*, exhibited cytotoxicity on JR4-Jurkat cells was related to accumulation of intracellular ROS, reduction of mitochondrial membrane potential, and cell apoptosis [20]. The convincing genetic/biochemical evidence has accumulated so far to show that taxol-mediated apoptosis solely relies on the intrinsic or the mitochondrial pathway [37].

Apoptosis is executed through mitochondrial-mediated intrinsic and cell death receptor-mediated extrinsic pathways, both of which converge on the cascade leading to activation of caspase proteases [63]. In the intrinsic pathway, cytochrome c releases from damaged mitochondria and causes apoptosome-dependent activation of caspase 9. This leads to the activation of the executioner caspase 3 [64], and eventually proteolytic inactivation of PARP [62]. In the extrinsic pathway, activated initiator caspase-8 is activated by death inducing signaling complex which then cleaves and activates caspase-3 and results in apoptotic cell death [65]. Our results demonstrated that fungal EDT increased the levels of cleaved, PARP in a dose-dependent manner, indicating that intrinsic pathway was involved in the process of fungal EDT-induced apoptosis in HepG2 cells. The intrinsic apoptosis pathway is largely caspase 9-dependent which further activates caspase-3 which has PARP as a substrate, among others. Cleavage of PARP facilitates cellular disassembly and serves as a marker of cells undergoing apoptosis [66].

The increased Bax/Bcl-2 ratio leads to ΔΨm collapse, cytochrome c release, caspase-3 activation, and eventually apoptosis [67]. In addition, pro-apoptotic protein Bax is closely associated with the control of mitochondrial membrane permeability and release of cytochrome c [68]. Our results showed that fungal EDT increased the levels of Bax (Pro apoptotic protein) decreased the levels of Bcl-2 (an anti-apoptotic protein) and cleavage of PARP together with signalling factor p38 in a dose-dependent manner, confirming that fungal EDT-induced apoptosis in HepG2 cells. Mitogen-Activated Protein Kinases (MAPKs) have been described as the major oxidative stress-sensitive signal transducing pathways [69] and serve as upstream signals for the initiation of apoptosis. JNK and p38 MAPK have been shown to get activated response to ROS generation and mitochondrial dysfunction, which are frequently associated with the induction of apoptosis [70]. Previous studies have reported natural compounds induced apoptosis in HepG2 cells through ROS mediated MAPK activation and mitochondrial dysfunction [71, 72]. Our results showed that fungal EDT significantly increased the level of p38 in a dose-dependent manner without affecting the expression of total proteins, indicating that these MAPK pathways were activated in the process of fungal EDT -induced apoptosis in HepG2 cells.

## Conclusions

This is the first report of 7-*epi*-10-deacetyltaxol (EDT) isolated form any microbial source. Our studies show that fungal EDT significantly inhibits HepG2 cells by arresting the cells in the G2/M phase of cell cycle, there by inducing apoptosis. The fungal EDT-induced apoptotic cell death in HepG2 cells through intrinsic pathway via triggering ROS generation and activating p38 MAPK pathway. This study provides an insight into the molecular mechanisms of fungal EDT-induced apoptosis in liver cancer cells and presents that fungal EDT as a novel promising agent for liver cancer treatment.

## Abbreviations

BLAST: Basic local alignment search tool
DCM: Dichloromethane
EDT: 7-*epi*-10-deacetyltaxol
PARP: Poly [ADP-ribose] polymerase
PBS: Phosphate-buffered saline
PCR: Polymerase chain reaction
PDA: Potato dextrose agar
TLC: Thin layer chromatography
